# Report of Departmental Committee on Sickness Benefit Claims

**Published:** 1915-01-09

**Authors:** 


### NATIONAL INSURANCE ACT DEVELOPMENTS.

### Report of Departmental Committee on Sickness Benefit Claims.

The Report of the Departmental Committee on Sickness Benefit Claims, which was published at the beginning of December last, is worthy of the closest possible attention by all who are interested in the subject of health insurance and the bearing of such insurance on the larger question of public health.

The Committee was appointed as long ago as August 22, 1913, the terms of reference being "to inquire into and report upon the alleged excessive claims upon and allowances by approved societies in England in respect of sickness benefit, and any special circumstances which may cause any such claims or allowances." Sir Claud Schuster, of the Insurance Commission, was chairman, and the Committee, which was a very strong one, included besides the chairman three other official representatives of the Insurance Commissioners, one of whom, Mr. J. S. Whitaker, also represented the medical profession. Four other members were doctors, and the remaining six members represented approved societies of diverse types.

The Committee began its labours in October 1913, and met twice a week to receive evidence, the sittings for this purpose numbering fifty-nine and extending until the end of May 1914. During this period no fewer than ninety-four witnesses were examined, the minutes of their evidence being published in three volumes with a separate index. The witnesses are classified in the Report as follows: (*a*) fifty-two represented approved societies, the choice being made so that practically every type of society was included; (*b*) twenty-six represented the medical profession, the practitioners being selected from widely differing areas, and not all of them being on the panels; (*c*) nine represented Insurance Committees; and (*d*) the remaining seven were witnesses who, while not engaged in connection with the administration of the Act, have made a special study of sickness insurance or of the effect of the National Insurance Act in operation. These particulars as to the terms of reference, the *personnel* of the Committee, and the representative character of the witnesses are important, as they invest the findings of the Committee with an authority which cannot be denied.

Before proceeding to consider the Report in some detail, it only remains to be stated that it was signed by all the members of the Committee. There are, however, four memoranda by separate members. One of these—namely, that signed by Miss Mary R. MacArthur—will call for special mention in due course.

The problem to be investigated was not strictly of an actuarial nature; in fact, the Committee was appointed when the Act had only been in operation for little more than twelve months, and payments on account of sickness benefit had only been made for a little over seven months, and consequently no figures of a detailed character, such as would be required for an actuarial investigation, were available. The task, therefore, was to examine the actual working of the administrative machinery of the Act as exemplified in the action of its several parts, rather than to investigate the financial results.

It was found to be necessary to consider the question from three different standpoints—namely, that of the insured persons as individuals, the doctors, and the approved societies. Moreover, as in the opinion of the Committee the framework of the Act is built up on sickness benefit, it was felt to be desirable to extend the scope of the inquiry so that the bearing of the administration of medical and maternity benefits upon the fundamental problem might be investigated.

### Effects of Double Insurance.

It is pointed out that the actuarial estimates which formed the basis of the financial provisions of the Act were based upon an average applicable to the insured population as a whole, distinguishing as between men and women, whereas the Act provided for the administration of the benefits through separate approved societies. Only in the very largest societies can it be assumed that the experience will approximate to the average of the country as a whole. The societies are of most diverse types, not only with regard to methods of administration, but also in regard to the character of their membership. There are societies, for instance, in which a careful selection of members was made, a medical examination being required before admission, while other societies, drawing their members exclusively from particular trades and without any examination, are composed of those who are subject to peculiarly hazardous risks as regards sickness experience. In these circumstances it is a foregone conclusion that there must inevitably be wide differences between the experiences of different societies, and this was fully evidenced by the witnesses who represented such societies.

Another point of considerable importance that is emphasised in the Report is the failure of Section 72 of the Act, which was designed to allow a reduction of the contributions paid by an existing member of a friendly society to the private funds of his society, and the transfer of the insurance from such private funds to the State section. In spite of the advantage which would have accrued to the funds of the friendly societies if this provision had been compulsorily adopted the action that was taken was negligible. As a result, so far as can be judged from the figures available, about 96 per cent. of the existing members of friendly societies who could have elected to reduce their contributions in the manner indicated have decided to pay the contributions under the National Insurance Act in addition to their former payments, thus increasing the amount receivable in the event of sickness. It therefore follows that a very large proportion of those insured persons who are members of approved societies formed by the old friendly societies, numbering in all nearly one-half of the total insured population, are insured against sickness by the private funds of their societies, and in consequence the extent of double insurance is considerable.

It has been suggested that this double insurance is in large measure the cause of excess claims for sickness benefit, as the sum insured in such circumstances may be a high proportion of, if it does not actually exceed, the average weekly earnings of the insured person, and may act as a temptation to malinger. On the other hand, various witnesses argued that in view of the heavy expenses connected with sickness the amount obtainable as benefit should not be less than the ordinary wage, and they held that it was only the thrifty and, consequently, healthy man who would pay the correspondingly heavy contributions required for such adequate insurance. The Committee found it very difficult to generalise on this subject of double insurance, but in view of the favourable experience of the societies established on the deposit system, which furnishes an inducement to refrain from drawing money on the private side, they appear to think that double insurance is to some extent at least a contributory cause of the excess claims for benefit. They do not think, however, that it would be possible to obtain legislative prohibition of insurance above a fixed limit, but suggest that the societies by making inquiries as to the amounts insured in the event of sickness with other societies and comparing the total with the average wage have the means in their own hand for restraining applications for membership of those who are over-insured, while they further point out that it is still possible for societies to adopt the provisions of Section 72 of the Act of 1911, already referred to.

### Prolonged Claims and Unsuspected Illness.

There are other causes of the excess sickness claims which were brought to the notice of the Committee. One of these was the novelty of the insurance. This particularly affected societies which were newly formed at the commencement of the operation of the Act. Again, there appears to have been evidence of an unwillingness to bring the period of incapacity to an end, and of difficulty in getting an insured person who has once declared on the funds to declare off.

In some instances there has been a disposition to make use of the sickness benefit while out of employment, while in certain cases the prolongation of claims appears to have been due to an intention to get the most out of the Act, pointing rather to an over-keenness of business instinct than any attempt at dishonest practices. The Committee summarise this section of the investigation by saying that "it would be idle and extravagant to base upon such an experience as we have had before us a general charge, either of malingering or of greed, against the insured population generally."

The excess of the claims for benefit above the expectation had in many cases to be admitted, and such excess was principally in connection with the insurance of women. The Committee consider that the excess is primarily due to the existence if not throughout the entire insured population, at any rate throughout certain classes and in certain grades, of much more sickness than we have been conscious of. "The evidence of medical practitioners," they say, "is overwhelmingly in support of the view that the effect of the Act has been to disclose, especially among industrial women, an enormous amount of unsuspected sickness and disease, and to afford treatment to many who have hitherto been without medical attendance during sickness." This condition may, however, only be temporary, for there are already indications that as a result of the rest obtained under the Act a better condition of health has in certain cases been attained than has been experienced for many years. It is anticipated that in time the general standard of health will tend to improve, and thus one of the principal objects of the Act—namely, the prevention (as well as the cure) of disease—will have been indirectly attained.

Space does not allow us to deal with the important section of the Report which is devoted to the consideration of the effect of the different forms of administration, local and central, upon the experience of the approved societies, but it is pointed out that there is a great danger that in the multiplicity of systems of government, with varying degrees of licence allowed to local officials, abuses may arise in the administration of the Act leading to the admission of improper claims or the rejection of just ones. It is sufficient to say in this connection that the Committee lay the strongest possible emphasis on the necessity for efficient investigation by the approved societies of the claims that are made upon them for the payment of benefits. A laxity on the part of the society—meaning here the responsible officials—leads inevitably to an excess of claims on the part of the insured member. One special feature that has to be noted is the importance attached to an efficient system of sick visitation, the absence of which has frequently been a contributory cause in leading to the admission of improper claims.

### Excess Sickness and Medical Practitioners.

The part taken by the doctor in relation to claims for sickness benefit is the important one of certifying that the insured person is "incapable of work." It is the duty of the panel doctor by virtue of his agreement to furnish such certificates as may be required for this purpose. It was never intended, however, that the production of a certificate of "incapacity," signed by a panel doctor, should relieve the society of its responsibility in the admission of claims for benefit. A good deal of the dissatisfaction which appears to have existed on the part of the approved societies with the certification appears to have arisen from the fact that a section of the doctors considered that their certificates should be accepted without question. The Committee, however, lay stress on the necessity for certification of the exact name of the disease, so far as known, from week to week.

Women's insurance we shall deal with later.

